# Dynamic Occlusion–Predictive Neural Network for Robust Roadside Multi-Vehicle Tracking

**DOI:** 10.3390/s26113529

**Published:** 2026-06-02

**Authors:** Shuai Wang, Yafei Wang, Bowen Wang, Chongfeng Wei, Hao Liu

**Affiliations:** 1 School of Mechanical Engineering, Shanghai Jiao Tong University, Shanghai 200240, China; wangshuai2020@sjtu.edu.cn (S.W.); 98.wangbowen@sjtu.edu.cn (B.W.); weichongfeng@gmail.com (C.W.); 2 The Beijing Intelligent Transport Development Center, Beijing 100053, China; hao.liu@jtw.beijing.gov.cn

**Keywords:** point cloud processing, roadside perception, multi-vehicle tracking, dynamic occlusion

## Abstract

**Highlights:**

**What are the main findings?**

- Dynamic Occlusion State Prediction via Transformer: We propose a Transformer-based framework that explicitly forecasts the future occlusion ratio of targets by modeling historical trends. By integrating this prediction as a dynamic weighting factor into the loss function, our model adaptively learns to mitigate the impact of varying occlusion severities, significantly enhancing tracking stability during continuous state changes.
- Physical-Constraint Reasoning via GNN: We develop a GNN-based module that leverages road occupancy and neighboring vehicle poses to infer the existence and motion patterns of targets within occluded regions. This module constructs a heterogeneous graph to determine scene physics, effectively linking fragmented trajectories by generating “virtual perception” states for invisible targets.

**What are the implications of the main findings?**

- Implication of Dynamic Occlusion State Prediction: Introducing predicted occlusion states into the optimization objective shifts the paradigm from passive reaction to proactive anticipation. This implies that explicitly modeling the dynamics of visibility is crucial for robust tracking, as it allows the system to maintain trajectory continuity even when targets undergo rapid and severe transitions between visible and occluded states.
- Implication of Physical-Constraint Reasoning: Inferring target existence through road occupancy and neighbor interactions demonstrates that scene priors can effectively substitute for missing sensory data. This creates a “reasoning-based tracking” capability that overcomes the physical limitations of roadside sensors, ensuring high-precision association and minimizing ID switches even in fully occluded or “blind” spots.

**Abstract:**

Despite their extended detection ranges and superior precision compared with onboard sensors, roadside perception systems suffer from severe occlusion artifacts in complex traffic, causing significant tracking failures and ID switches. To address this, we propose a novel Dynamic Occlusion–Predictive Neural Network tailored to challenging roadside environments. First, we introduce a Transformer-based Dynamic Occlusion State Predictor to explicitly model the temporal evolution of occlusion. Unlike traditional tracking methods, this module continuously forecasts future occlusion ratios for each target by analyzing historical occlusion patterns. Critically, these predictions are integrated into the tracking framework as dynamic weighting factors in the loss function, enabling the model to adaptively penalize tracking errors based on the predicted occlusion severity and significantly enhancing robustness against dynamic occlusion scenarios. Second, leveraging the predicted occlusion states, we propose a GNN-based Spatial Reasoning Module to address trajectory fragmentation. This module constructs a heterogeneous graph integrating road occupancy information and neighboring vehicle poses to infer the existence and motion patterns of targets within occluded regions. By analyzing scene-level physical constraints, it generates motion predictions for invisible targets and links these inferred states to fragmented trajectories, ensuring temporally continuous tracking even during prolonged visual occlusions. Experiments on the DAIR-V2X and our self-collected roadside dataset show that our framework outperforms state-of-the-art methods in precision and robustness, achieving a 5.1% MOTA gain over the best baseline. This advantage peaks under high occlusion, where preserving ID continuity and minimizing failures validates its efficacy for real-world roadside multi-target tracking.

## 1. Introduction

Roadside perception systems equipped with advanced sensor arrays offer extended detection ranges and superior precision compared with onboard sensors, positioning them as a cornerstone for intelligent transportation systems [1,2,3]. However, this “bird’s-eye” advantage is severely limited in long-range, high-density traffic scenarios, where the unique geometry of roadside views introduces distinct challenges. Unlike onboard perspectives, roadside sensors suffer from extreme perspective distortions at a distance, where vehicles appear as sparse, indistinguishable clusters. This leads to a highly dynamic occlusion environment: targets frequently transition between partial and full occlusion states due to the dense packing of traffic and the specific viewing angle. These rapid fluctuations in visibility, combined with feature sparsity caused by long-range distances, create a noisy observation space where standard tracking filters struggle to maintain continuity, leading to a significant degradation in precision and frequent identity (ID) switches [4,5].

The core difficulty in robust roadside tracking stems from the ill-posed nature of tracking under dynamic and total occlusion. First, the temporal instability of occlusion states, where a target’s visibility ratio changes drastically from frame to frame, makes it difficult for traditional models to distinguish between actual trajectory deviations and observation noise. Without a mechanism to explicitly anticipate these changes, tracking models often fail to adapt their confidence levels dynamically. Second, in scenarios of total occlusion caused by dense traffic, the target’s motion priors (velocity, acceleration) become completely unobservable. Traditional methods, which rely heavily on historical motion states, fail here because they lack the contextual reasoning capability to infer the missing motion patterns from the surrounding environment (e.g., utilizing the “social forces” of neighboring vehicles or the geometric constraints of the road). Consequently, preserving target identity through these periods of invisibility remains a formidable challenge, often resulting in irreversible track fragmentation [6,7].

While concerted efforts have been directed toward mitigating occlusion, existing state-of-the-art methods struggle to generalize in roadside scenarios. Recent Transformer-based trackers [8,9,10] excel at global sequence modeling but often treat occlusion as a passive “missing data” problem, relying on interpolation rather than active reasoning. While effective in onboard settings, they lack a mechanism to explicitly model the dynamic evolution of occlusion states, rendering them vulnerable to the rapid visibility fluctuations typical of roadside views. Conversely, Graph Neural Network (GNN) approaches [11,12,13] model inter-object interactions but typically focus on social grouping or simple collision avoidance. They frequently overlook the physical constraints of the road geometry (e.g., lane occupancy), which are critical for inferring the state of a target when visual features are sparse. Consequently, without explicitly leveraging road topology or predicting future occlusion severity, these methods fail to maintain robust identity consistency during severe and prolonged occlusion events [14,15].

In this paper, to bridge these gaps, we propose a Dynamic Occlusion–Predictive Neural Network tailored for robust roadside multi-vehicle tracking. Unlike reactive paradigms that merely compensate for detection failures post hoc, our framework treats occlusion as a predictable physical state. We introduce a dual-component architecture designed to handle the specific challenges of the roadside perspective: (1) Proactive State Prediction: We employ a Transformer-based Occlusion State Predictor that not only captures long-range temporal dependencies to filter stochastic noise but also explicitly forecasts future occlusion ratios. This prediction is fed back as a dynamic loss weight, allowing the model to adaptively prioritize targets at risk of disappearing. (2) Physical-Constraint Reasoning: We develop a GNN-based Spatial Reasoning Module that goes beyond simple social pooling. By constructing a heterogeneous graph incorporating road occupancy and geometric constraints, the module infers the existence and motion of invisible targets based on the physical plausibility of the scene. This transforms the ill-posed problem of missing priors into a solvable inference task, ensuring precise association even under complete visibility loss.

The main contributions of this work are summarized as follows:

(1) Dynamic Occlusion State Prediction via Transformer with Feedback-Driven Loss Optimization: We propose a novel Transformer-based framework that explicitly models the temporal evolution of occlusion. Unlike standard tracking methods that treat occlusion as a static label, our approach forecasts the future occlusion ratio by analyzing historical trends across frames. Crucially, we introduce a mechanism where this predicted state is fed back into the tracking framework as a dynamic weighting factor within the loss function. This allows the model to adaptively adjust the penalty for prediction errors based on the predicted severity of occlusion, significantly enhancing the network’s robustness against targets undergoing continuous and dynamic state changes.
(2) Scene-Aware Virtual Perception via GNN for Existence Inference and Trajectory Linking: Building upon the predicted occlusion states, we develop a Graph Neural Network (GNN) module designed for reasoning under uncertainty. This module constructs a heterogeneous graph incorporating road occupancy and neighboring vehicle poses to explicitly infer the existence of targets within occluded regions. By analyzing these physical constraints, the model predicts the latent motion patterns of invisible targets and generates “virtual features” to bridge trajectory gaps. This approach effectively transforms the tracking problem from simple feature matching to physics-constrained inference, ensuring continuous trajectory linking and minimizing ID switches even when targets are completely obscured.
(3) State-of-the-Art Performance on Benchmark Datasets: We conduct extensive evaluations using the large-scale DAIR-V2X dataset and a self-collected complex urban dataset. The quantitative results demonstrate that our method achieves a MOTA of 92.6% on DAIR-V2X and 80.6% on the self-collected complex dataset, outperforming current state-of-the-art methods. Notably, our approach reduces identity switches (IDSs) by 37% and trajectory fragmentation (FRAG) by 25% compared with the second-best method (AGO-Net), validating the superior robustness of our temporal–spatial fusion architecture in maintaining track continuity.

**Figure 1:**
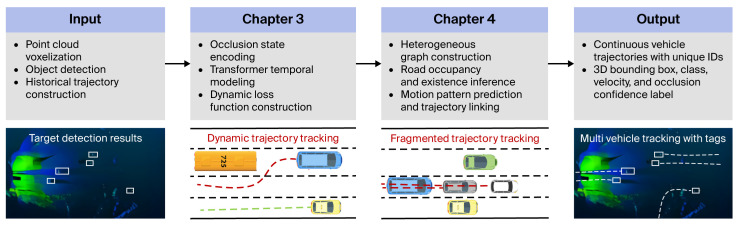
Illustration of the proposed dynamic occlusion–predictive tracking framework. The architecture is divided into the core methodologies of Section 3 and Section 4. Section 3 utilizes a Transformer-based encoder to model temporal dependencies and construct a dynamic loss function for handling stochastic noise. Section 4 employs a Graph Neural Network (GNN) to construct a heterogeneous graph for inferring road occupancy and predicting motion patterns during occlusion. The final output provides robust 3D bounding boxes, target parameters, and identity consistency for multi-vehicle tracking.

The remainder of this paper is organized as follows, with the overall framework illustrated in Figure 1. Following the introduction of related work in Section 2, the core methodology is detailed in Section 3 and Section 4. As depicted in the pipeline, the system takes voxelized point clouds and historical trajectories as input. Section 3 presents the Transformer-based global temporal modeling module. This component addresses the temporal instability of occlusion by encoding the historical trajectory of each target, predicting the dynamic evolution of the occlusion state, and constructing a dynamic loss function to suppress stochastic noise caused by abrupt maneuvers, ensuring robust tracking continuity (as shown in the “Dynamic trajectory tracking” panel). Subsequently, Section 4 introduces the GNN-based Interaction Module designed to recover fragmented trajectories. By constructing a heterogeneous graph that incorporates road occupancy and neighboring vehicle poses, this module infers the existence and latent motion patterns of occluded targets. This mechanism effectively links trajectory fragments under severe occlusion (illustrated in the “Fragmented trajectory tracking” panel), transforming ambiguous associations into a structured inference task. Finally, Section 5 validates the proposed framework through extensive experiments, demonstrating significant improvements in tracking precision and ID consistency. Section 6 concludes the paper and discusses future research directions.

## 2. Related Work on Occlusion-Aware Tracking Methods

In this section, we review the evolution of 3D multi-object tracking, establishing the theoretical foundations of this work. We scrutinize the dominant paradigms, specifically detection-driven association and Bayesian filtering, and their limitations in modeling non-linear motions and multi-modal uncertainties. We highlight how the unimodal Gaussian assumption in traditional filters degrades performance in complex scenarios and how recent hybrid architectures bridge probabilistic inference with deep learning. Building upon this analysis, we address the critical bottleneck of occlusion-aware tracking. While existing methods leverage temporal smoothing, prediction, and semantic priors to mitigate fragmentation, they lack a unified mechanism for proactive occlusion reasoning. This gap in predictive state estimation is the motivation for the proposed framework that integrates spatial topologies with temporal dynamics to forecast occlusion states.

### 2.1. 3D Multi-Object Tracking Paradigms

3D multi-object tracking (MOT) methodologies are generally categorized into detection-driven paradigms and Bayesian state estimation frameworks [16,17]. While detection-driven approaches utilizing 3D CNNs or PointNet-like architectures have achieved significant progress in feature extraction [18], they often treat tracking as a static association problem, neglecting the temporal continuity essential for handling roadside occlusion. Conversely, Bayesian filtering frameworks, such as Kalman filters (KFs) and their non-linear extensions (EKF, UKF), model target dynamics by propagating state means and covariances [19].

However, a critical limitation of these parametric Bayesian methods lies in their strict unimodal Gaussian assumption. In roadside scenarios characterized by severe occlusion and long-range perspective distortion, the target’s state distribution is inherently multi-modal (e.g., a vehicle could be behind an obstacle or simply missed by the sensor). The unimodal assumption fundamentally precludes accurate modeling of these complex uncertainties [20]. Although Sequential Monte Carlo (SMC) methods (Particle Filters) were introduced to approximate arbitrary distributions, they incur exponential computational complexity and suffer from particle degeneracy in high-density traffic [21,22].

Recently, the field has shifted towards integrating deep learning with probabilistic inference. For instance, in 2025, K Chen et al. proposed an uncertainty-based roadside point cloud detection and tracking method to enhance perceptual robustness [23]. While this represents a significant step forward in acknowledging sensor noise, it primarily focuses on spatial uncertainty at the detection level and lacks a mechanism to model the temporal evolution of occlusion, failing to address the specific challenge of rapid visibility changes caused by the unique “bird’s-eye” perspective of roadside sensors. Consequently, the existing methods remain reactive, struggling to maintain trajectory continuity when targets transition abruptly between visible and fully occluded states.

### 2.2. Occlusion-Aware Tracking Methods

Robust tracking in occluded scenarios remains a bottleneck, primarily due to feature sparsity and ambiguous data association in crowded environments [24,25,26]. The existing literature addresses these challenges through three main paradigms, each with inherent limitations in the context of roadside perception.

First, temporal modeling approaches leverage Recurrent Neural Networks (RNNs) like LSTMs to capture motion dynamics [27,28,29]. However, their sequential processing nature limits parallelization and prevents them from capturing the global spatial–temporal context required to resolve complex occlusions. Second, semantic-aware methods incorporate priors such as lane geometry or social forces [30,31]. While helpful, these approaches often treat environmental constraints as static regularizers rather than dynamic predictors, while relying on observable cues and struggling to infer latent motion patterns when targets are fully occluded.

More recently, communication-aware approaches have emerged to tackle environmental challenges. Notably, in 2025, A Rehman et al. proposed an uncertainty-based roadside point cloud detection and tracking method that utilizes intelligent reflecting surfaces to mitigate signal blockage in Non-Line-of-Sight (NLOS) conditions [32]. While effective for communication connectivity, such physical-layer solutions do not resolve the perceptual ambiguity in computer vision tasks and do not provide the semantic understanding or trajectory prediction capabilities necessary for a roadside perception system to “see through” occlusions via algorithmic inference.

Crucially, a significant gap remains in proactive occlusion modeling. The current methods are largely reactive, compensating for missed detections after they occur, and there is a lack of frameworks that can explicitly predict the dynamic evolution of occlusion states (e.g., predicting when a vehicle will emerge from behind a bus) by jointly leveraging temporal history and spatial interactions. To bridge this gap, our work departs from passive observation by fusing a Transformer-based temporal encoder with a GNN-based interaction module, enabling the proactive forecasting of occlusion states and robust trajectory recovery.

## 3. Dynamic Occlusion State Prediction via Spatiotemporal Transformer

**Figure 2:**
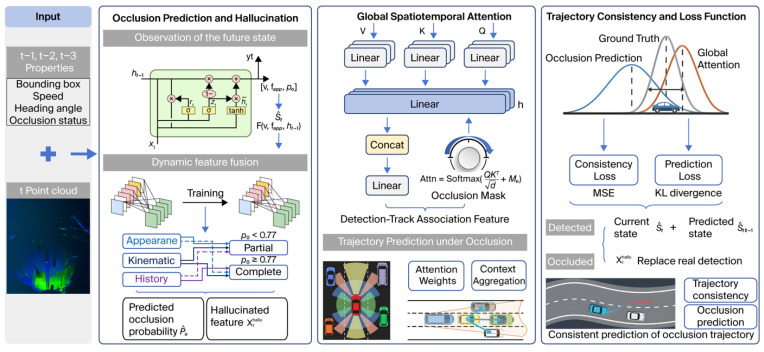
Overview of the proposed Occlusion–Predictive Spatiotemporal Transformer. The framework comprises three core components aligned with our methodology: (1) Occlusion State Prediction (Section 3.1): Utilizes historical observations to predict occlusion probabilities and employs a semi-supervised temporal consistency mechanism to hallucinate features for partially or fully occluded targets; (2) Global Spatiotemporal Attention (Section 3.2): Models long-range dependencies via linear-transformed queries, keys, and values, integrating an occlusion mask to refine detection-track association in complex scenarios; and (3) Trajectory Consistency (Section 3.3): Enforces robust tracking through space-time smoothing, optimizing trajectory alignment via MSE-based consistency loss and KL divergence-based occlusion prediction loss.

The fundamental challenge of 3D MOT under occlusion is bridging noisy observations with continuous physical motion. Traditional methods fail to capture multi-modal scenarios or maintain identity consistency during occlusion. In this section, we introduce a predictive framework that treats occlusion as a physically constrained state transition, integrating a Spatiotemporal Transformer with Occlusion-Aware Attention to model temporal dependencies and a dynamic loss function to enforce motion inertia. In this section, we present the proposed Occlusion-Predictive Spatiotemporal Transformer, whose overall architecture and information flow are illustrated in Figure 2. Aligned with the methodology detailed in this chapter, the framework is built upon three core components: (1) Occlusion State Prediction (Section 3.1), which leverages historical observations to estimate occlusion probabilities and employs a semi-supervised temporal consistency mechanism to hallucinate features for occluded targets; (2) Global Spatiotemporal Attention (Section 3.2), designed to capture long-range dependencies via linear-transformed queries, keys, and values, while integrating an occlusion mask to refine detection-track association in complex scenarios; and (3) Trajectory Consistency (Section 3.3), which enforces robust tracking through space-time smoothing by optimizing trajectory alignment via an MSE-based consistency loss and a KL divergence-based occlusion prediction loss. This synergistic design provides a robust foundation for addressing occlusion challenges, with the specific mechanisms of each module elaborated in the following subsections.

### 3.1. Problem Formulation and Occlusion State Encoding

#### 3.1.1. Occlusion State Transition

We define the occlusion state $s_{t}\in [0,1]$ at time t, where $s_{t}\approx 0$ indicates full visibility and $s_{t}\approx 1$ indicates full occlusion. To capture the non-linear motion dynamics that linear Kalman filters cannot represent, we employ a Long Short-Term Memory (LSTM) network. Let $x_{t}=\left[p_{t},v_{t},a_{t}\right]$ be the input vector at time t, comprising kinematic features (position p, velocity v) and appearance features a. The LSTM unit updates its internal hidden state $h_{t}$ and cell state $c_{t}$ based on the previous hidden state $h_{t-\mathrm{text}\;1}$ and the current input $x_{t}$. Let $\left[h_{t-1},x_{t}\right]$ denote the concatenation of the two vectors. The gate activations are computed as follows:

(1) $$i_{t}=\sigma \left(W_{i}\left[h_{t-1},x_{t}\right]+b_{i}\right)\;f_{t}=\sigma \left(W_{f}\left[h_{t-1},x_{t}\right]+b_{f}\right)\;o_{t}=\sigma \left(W_{o}\left[h_{t-1},x_{t}\right]+b_{o}\right)\;{\tilde{C}}_{t}=tanh\left(W_{C}\left[h_{t-1},x_{t}\right]+b_{C}\right)$$

Here, $i_{t},\;f_{t}$, and $o_{t}$ denote the input, forget, and output gates, respectively. σ is the sigmoid activation function, and ⊙ represents the Hadamard product. The forget gate $f_{t}$ plays a crucial role in enforcing temporal inertia by determining how much of the previous cell state $C_{t-1}$ (historical context) is retained. By learning to keep $f_{t}\approx 1$ during stable motion, the model resists rapid, noisy fluctuations in the occlusion state. W and b are learnable weight matrices and bias vectors, respectively, while σ denotes the sigmoid function. The cell state $C_{t}$ and hidden state $h_{t}$ are then updated as follows:

(2) $$C_{t}=f_{t}⊙C_{t-1}+i_{t}⊙{\tilde{C}}_{t}\;h_{t}=o_{t}⊙tanh\left(C_{t}\right)$$

The predicted occlusion probability ${\hat{s}}_{t}$ is derived from the hidden state $h_{t}$:

(3) $${\hat{s}}_{t}=\sigma \left(W_{s}\cdot h_{t}+b_{s}\right)$$

#### 3.1.2. Semi-Supervised Learning with Inertia Regularization

A critical challenge is the scarcity of fully annotated occlusion labels in real-world datasets. To address this, we formulate a semi-supervised learning objective. The total loss function $L_{\mathrm{total}}$ consists of a supervised classification term $L_{\mathrm{cls}\;}$ and a novel inertia regularization term $L_{\mathrm{inert}}$:

(4) $$L_{\mathrm{total}\;}=L_{\mathrm{cls}\;}\left({\hat{s}}_{t},s_{t}^{gt}\right)+\lambda L_{\mathrm{inert}\;}$$

The inertia loss $L_{\mathrm{inert}}$ penalizes the L2-norm of the difference between consecutive predictions:

(5) $$L_{\mathrm{inert}\;}={\left\|{\hat{s}}_{t}-{\hat{s}}_{t-1}\right\|}^{2}$$

Physically, this enforces the principle that motion continuity–occlusion states should evolve smoothly unless interrupted by a drastic event (e.g., a sudden cut-in). The hyperparameter λ balances the tradeoff between responsiveness and stability.

#### 3.1.3. Temporal Feature Hallucination via Visible Feature Bank

When an object is fully occluded ($s_{t}\approx 1$), direct feature extraction fails. To maintain the “identity anchor,” we propose a Temporal Feature Hallucination mechanism. Unlike naive methods that carry over the last known feature (leading to ID drift), we synthesize the feature $a_{t}^{\mathrm{syn}}$ by attending to a Visible Feature Bank (VFB).

The VFB is implemented as a First-In–First-Out (FIFO) queue, $B=\left\{\left(a_{i},l_{i},t_{i}\right)\right\}$, storing appearance features $a_{i}$, lane IDs $l_{i}$, and timestamps $t_{i}$ of all visible neighbors.

To construct the synthesized feature, we first select candidate neighbors N within the same lane ($l_{i}=l_{\mathrm{target}}$) and within a spatial threshold $d_{\max}$:

(6) $$N=\left\{i\in B|\left\|p_{i}-p_{\mathrm{target}\;}\right\|<d_{max}\wedge l_{i}=l_{\mathrm{target}}\right\}$$

The synthesized feature is then computed as a weighted average of the candidates in N, where weights are determined by spatial proximity:

(7) $$a_{t}^{syn}=\frac{1}{Z}\sum_{i\in N}exp\left(-\left\|p_{i}-p_{target}\right\|\right)\cdot a_{i}$$

This mechanism allows the tracker to infer the likely appearance of a hidden object based on the flow of surrounding traffic, ensuring robust re-identification after occlusio.

### 3.2. Global Temporal Modeling with Occlusion-Aware Attention

To overcome the limitations of frame-by-frame association, which is prone to local ambiguity under severe occlusion, we propose a global temporal modeling (GTM) module, which operates on a sliding temporal window T={t−N,…,t} to establish long-range dependencies. The core idea is to construct a global affinity matrix that links detections across time, enabling the tracker to “see through” occlusions by leveraging historical context.

#### 3.2.1. Unified Spatiotemporal Embedding

First, we project heterogeneous inputs into a unified d-dimensional embedding space. Let $x_{t}^{\left(i\right)}$ denote the input feature for the i-th object at time t, which encompasses kinematic states $p_{t},v_{t}$, appearance features $a_{t}$, and the occlusion probability ${\overset{ˆ}{s}}_{t}$ predicted in Section 3.1. We apply a linear projection $W_{e}$ followed by sinusoidal positional encoding PE(⋅) to inject temporal order:

(8) $$z_{t}^{\left(i\right)}=W_{e}\cdot x_{t}^{\left(i\right)}+PE(t)$$

Here, PE(t) ensures that the model can distinguish objects with similar appearances but different motion phases (e.g., accelerating vs. decelerating).

#### 3.2.2. Occlusion-Aware Self-Attention

Standard self-attention computes affinities based solely on feature similarity, which fails when appearance features are corrupted by occlusion. We introduce an Occlusion-Aware Attention (OAA) mechanism that dynamically modulates the attention weights based on the predicted occlusion state ${\hat{s}}_{t}$ (from Section 3.1).

Given the Query Q, Key K, and Value V matrices derived from z, the attention score is computed as follows:

(9) $$\;\mathrm{Attention}\;(Q,K,V)=Softmax\left(\frac{QK^{T}}{\sqrt{d_{k}}}+M\right)V$$

The masking matrix $M\in R^{N\times N}$ encodes the physical constraint that occluded objects are unlikely to interact with distant objects. Specifically, we define the element $M_{ij}$ as follows:

(10) $$M_{ij}=\left\{\begin{array}{ll} -1\times 10^{9} & \;\mathrm{if}\;{\hat{s}}_{t_{i}}>\tau \;\mathrm{and}\;{\left\|p_{i}-p_{j}\right\|}_{2}>\delta \\ 0 & \;\mathrm{otherwise}\; \end{array}\right.$$

where τ is the occlusion threshold (e.g., 0.5) and δ is the spatial threshold (e.g., 10 m). ${\left\|p_{i}-p_{j}\right\|}_{2}$ denotes the Euclidean distance between object i and j. By adding a large negative value ($-1\times 10^{9}$) to the attention logits, we effectively prune the attention links between heavily occluded targets and nonadjacent candidates, forcing the model to rely on local motion coherence rather than noisy global features.

#### 3.2.3. Global Trajectory Association

The output of the Transformer encoder provides a refined feature representation $z_{t}^{(i)*}$ that encodes the global spatiotemporal context. We construct a bipartite graph between objects at time t−1 and t using a cosine similarity metric:

(11) $$A_{ij}=CosSim\left(z_{t-1}^{(i)*},z_{t}^{(j)*}\right)$$

This affinity matrix A serves as the input for the global optimization problem solved in Section 3.3. By integrating occlusion state priors into the attention mechanism, the model can maintain robust tracklets even when objects disappear for multiple frames.

### 3.3. Dynamic Loss Construction for Trajectory Stability

To train the proposed Spatiotemporal Transformer (Section 3.2) and ensure that the predicted trajectories adhere to physical laws, we construct a composite loss function. This loss function serves two purposes: it supervises the occlusion state prediction to handle missing detections and optimizes the embedding space to facilitate robust data association.

#### 3.3.1. Occlusion-Aware Prediction Loss

Building upon the formulation in Section 3.1, we define the total loss $L_{\mathrm{total}\;}$ as a weighted sum of the prediction error and a temporal regularization term. The prediction error ensures that the network outputs correct occlusion probabilities, while the regularization term enforces motion inertia.

(12) $$L_{\mathrm{total}\;}=\underset{\mathrm{Prediction}\;\mathrm{Error}\;}{\underbrace{{\left\|s_{t}-{\hat{s}}_{t}\right\|}_{2}^{2}}}+\lambda \underset{\mathrm{Second}\text{-}\mathrm{order}\;\mathrm{Regularization}\;}{\underbrace{{\left\|{\hat{s}}_{t}-2{\hat{s}}_{t-1}+{\hat{s}}_{t-2}\right\|}_{2}^{2}}}$$

Here, the second-order difference term (akin to jerk in physics) replaces the first-order term in the draft to better penalize sudden, unrealistic changes in visibility status. The hyperparameter λ controls the smoothness of the predicted state transition. This formulation directly addresses the challenge of “sudden disappearance” by discouraging the network from predicting transient occlusions unless strongly supported by evidence.

#### 3.3.2. Multi-Modal Association Metric

To resolve ambiguities in the global affinity matrix A (Section 3.2), we define a joint similarity metric that combines geometric, kinematic, and semantic cues. Unlike single-frame methods, our metric is computed over the spatiotemporal window to leverage historical context. The final affinity score $A_{ij}$ between detection i at time t−1 and detection j at time t is calculated as follows:

(13) $$A_{ij}=\alpha \cdot CosSim\left(f_{i},f_{j}\right)+\beta \cdot IoU\left(b_{i},b_{j}\right)+\gamma \cdot Mahal\left(x_{i},x_{j}\right)$$

where

Cosine similarity (CosSim) measures appearance consistency in the feature space, crucial for reidentifying objects after long-term occlusion.

Intersection over Union (IoU) measures the overlap of 2D bounding boxes, enforcing spatial proximity.

Mahalanobis distance (Mahal) measures the statistical deviation of kinematic states (position, velocity), weighted by the covariance matrix P from the Kalman filter:

(14) $$Mahal\left(x_{i},x_{j}\right)=\sqrt{{\left(x_{i}-x_{j}\right)}^{T}P^{-1}\left(x_{i}-x_{j}\right)}$$

The weights α,β,γ are learned during training to balance the contribution of each modality.

#### 3.3.3. Global Trajectory Optimization

Given the affinity matrix A, we solve for the optimal association $M^{*}$ using the Hungarian algorithm to minimize the global matching cost:

(15) $$M^{*}=\underset{M}{argmin}\sum_{i,j}\left(1-A_{ij}\right)\cdot M_{ij}$$

After association, we apply a Kalman update to refine the state estimate ${\overset{ˆ}{x}}_{t}$. For occluded targets (where no detection is associated), we retain the predicted state $x_{t}^{-}$ without measurement update, thereby maintaining trajectory stability through the occlusion period.

This dynamic loss construction ensures that the tracker prioritizes physically plausible trajectories while remaining robust to noisy observations.

## 4. Physical-Constraint Graph Reasoning for Interaction Modeling

**Figure 3:**
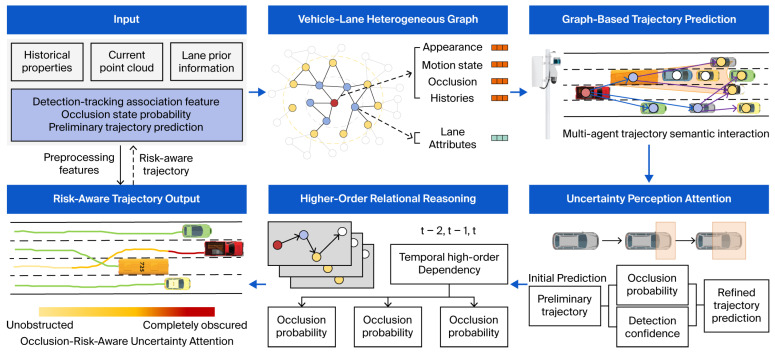
Overview of the proposed Physical-Constraint Graph Reasoning framework. The architecture consists of three progressive stages: (1) Heterogeneous Graph Construction with Roadside Geometric Priors (Section 4.1): Integrates vehicle detection features with lane prior information to establish a structured vehicle–lane heterogeneous graph, explicitly modeling geometric constraints; (2) Social–Geometric Message Passing Mechanism (Section 4.2): Performs higher-order relational reasoning on the graph to capture multi-agent semantic interactions and temporal dependencies; and (3) Uncertainty-Aware Existence Inference and Association (Section 4.3): Refines preliminary trajectory predictions by evaluating occlusion probabilities and detection confidence, ensuring robust association even under severe occlusion. The system ultimately outputs risk-aware trajectories that incorporate physical constraints and uncertainty estimation.

While temporal prediction provides a foundation for handling occlusion, it often neglects the rigid physical constraints imposed by the environment and social interactions. To address this limitation, we propose a Physical-Constraint Graph Reasoning framework that explicitly models the heterogeneous relationships between vehicles and the roadside infrastructure. This section formulates tracking as a relational inference problem on a dynamic graph, where geometric priors guide the message passing to ensure physical plausibility. Figure 3 illustrates the overall pipeline of our proposed Physical-Constraint Graph Reasoning framework, which systematically bridges low-level perception features with high-level trajectory planning. Unlike standard approaches, this architecture adopts a progressive reasoning strategy: it first constructs a Vehicle-Lane Heterogeneous Graph (Section 4.1) to embed rigid road geometric priors into the scene representation. Building on this structured graph, the model performs Higher-Order Relational Reasoning (Section 4.2) to capture complex social interactions and temporal dependencies among agents. Finally, to address the inherent ambiguity in occluded scenarios, an Uncertainty Perception Attention mechanism (Section 4.3) is introduced to dynamically refine predictions based on detection confidence. This end-to-end process ensures that the final output is not only socially compliant but also robust against severe occlusions, yielding risk-aware trajectories as detailed in the subsequent sections.

### 4.1. Heterogeneous Graph Construction with Multi-Source Constraints

To bridge the gap between dynamic object tracking (Section 3) and static environmental constraints, we construct a heterogeneous graph, G=(V,E), that explicitly models the interaction between vehicles and the road infrastructure. This graph serves as the structural foundation for relational reasoning, where nodes represent entities and edges encode physical and social constraints.

#### 4.1.1. Node Definition and Feature Initialization

The node set V consists of two disjoint subsets: dynamic vehicle nodes $V_{v}$ and static lane nodes $V_{ı}$. For each vehicle node $v_{i}\in V_{v}$, the initial feature embedding ${h_{i}}^{\left(0\right)}$ is formulated by fusing the refined features output from the Spatiotemporal Transformer (Section 3.2) with explicit geometric priors:

(16) $$h_{i}^{\left(0\right)}=f_{i}^{app}⊕f_{i}^{dyn}⊕f_{i}^{geo}$$

Here, ⊕ denotes concatenation. The components are defined as follows:

Appearance Feature ($f_{i}^{\mathrm{app}}$): This term is extracted from the ROI-pooled visual feature map, representing the texture and shape identity.

Dynamic Feature ($f_{i}^{\mathrm{dyn}}$): This term is derived from the kinematic state (velocity v, acceleration a) estimated in Section 3.3, providing motion context.

Geometric Prior ($f_{i}^{geo}$): This term anchors the node to the physical world. It is defined as $f_{‡}^{geo}=[\kappa ,\Delta \theta ]$, where κ is the local lane curvature and Δθ is the angular deviation between the vehicle’s heading and the lane tangent. This explicit encoding ensures that, even under severe occlusion (where $f_{i}^{app}$ may be noisy), the node retains a strong positional identity relative to the road structure.

#### 4.1.2. Edge Construction with Physical Thresholds

The edge set E is categorized into intra-type edges $E_{vv}$ (vehicle–vehicle) and inter-type edges $E_{vl}$ (vehicle–lane). The construction of these edges is governed by physical laws and geometric topology, addressing the concern regarding “threshold stability.”

Vehicle–Vehicle Edges ($E_{vv}$): We connect nodes $v_{i}$ and $v_{j}$ if their interaction is physically plausible. Instead of a fixed heuristic, the distance threshold δ is derived from a safety envelope model based on relative velocity:

(17) $$E_{vv}=\left\{\left(v_{i},v_{j}\right)|\left\|p_{i}-p_{j}\right\|\leq \delta \left(\Delta v_{ij}\right)\right\}$$

where $\delta \left(\Delta v_{ij}\right)=\left\|\Delta v_{ij}\right\|\cdot \tau +d_{min}$. Here, τ is the reaction time constant, and $d_{min}$ is the minimum stopping distance. This dynamic thresholding ensures graph sparsity and physical plausibility, preventing the inclusion of irrelevant long-range interactions.

Vehicle–Lane Edges ($E_{vl}$): We establish an edge if the vehicle is topologically associated with the lane. Let $p_{\mathrm{lat}}$ be the lateral offset. We define these edges as follows:

(18) $$E_{vl}=\left\{\left(v_{i},l_{j}\right)||p_{\mathrm{lat}\;}|\leq \epsilon \right\}$$

where ϵ is set to half the average lane width plus a small tolerance to account for localization noise. This strict geometric constraint enforces that the relational reasoning is bounded by the physical structure of the road.

#### 4.1.3. Edge Feature Encoding

Each edge $e_{ij}\in E$ is attributed a feature vector $e_{ij}$ that encodes the nature of the interaction. For $E_{vv}$, $e_{ij}$ includes relative position Δp, relative velocity Δv, and a social flag $s_{ij}$. For $E_{vl}$, $e_{ij}$ encodes the lateral offset $p_{\mathrm{lat}\;}$ and angular deviation Δθ. This heterogeneous graph construction provides a rigorous mathematical foundation for the subsequent Social–Geometric Message Passing (Section 4.2).

### 4.2. Social–Geometric Message Passing Mechanism

To address the limitations of linear motion assumptions (as discussed in Section 3.3) and to model the distinct nature of interactions, we propose a Social–Geometric Message Passing (SG-MP) mechanism. Unlike standard GNNs that use homogeneous aggregation, our method explicitly decouples Social Interactions (vehicle-to-vehicle) from Geometric Constraints (vehicle-to-lane). This module computes a motion residual Δx that corrects the kinematic prior, effectively providing a non-linear adaptation to the USV-specific dynamics.

#### 4.2.1. Social Interaction via Relative Dynamic Encoding

For vehicle-to-vehicle edges $E_{vv}$, the message must capture the reactive behavior between agents. We define the message $m_{ij}^{wv}$ as a function of the relative kinematic state, which serves as a proxy for collision risk and following behavior. Let $\Delta p_{ij}$ and $\Delta v_{ij}$ denote the relative position and velocity encoded in the edge features (Section 4.1). The message is computed as:

(19) $$m_{ij}^{vv}=W_{1}\cdot \sigma \left(W_{2}\left[h_{i}^{\left(l\right)}⊕h_{j}^{\left(h\right)}⊕\Delta p_{ij}⊕\Delta v_{ij}\right]\right)$$

where $h_{i}^{\left(l\right)}$ is the receiver’s state, $h_{j}^{\left(l\right)}$ is the sender’s state, and σ is the ReLU activation. This formulation forces the network to learn the relative dynamic pattern (e.g., deceleration due to a closer neighbor), directly compensating for the linear constant-velocity assumption in the tracking prior.

#### 4.2.2. Geometric Constraint via Topological Projection

For vehicle-to-lane edges $E_{vl}$, the message enforces the physical structure of the environment. Based on the geometric priors defined in Section 4.1 ($p_{\mathrm{lat}}$: lateral offset, Δθ: heading deviation), we compute a corrective message that acts as a “virtual spring” pulling the vehicle towards the lane center:

(20) $$m_{ik}^{vl}=W_{3}\cdot tanh\left(W_{4}\left[h_{i}^{\left(l\right)}⊕h_{k}⊕p_{lat}⊕\Delta \theta \right]\right)$$

Here, $h_{k}$ represents the static embedding of the lane node. By incorporating $p_{\mathrm{lat}\;}$ and Δθ explicitly, this term provides a strong topological constraint that stabilizes the trajectory estimation when visual features are noisy, effectively replacing the need for a complex non-linear dynamics model with a geometric potential field.

#### 4.2.3. Gated Fusion and Residual Update

To ensure training stability and prevent gradient explosion (addressing the concern on “stability analysis”), we employ a Gated Recurrent Unit (GRU) to fuse the incoming messages. The final update rule for node i at layer (l+1) is defined as:

(21) $$h_{i}^{\left(l+1\right)}=GRU(h_{i}^{\left(l\right)},\underset{\mathrm{Aggregated}\;\mathrm{Social}\;\mathrm{Context}\;}{\underbrace{\sum_{j\in N_{0}}m_{ij}^{vp}}}+\underset{\mathrm{Aggregated}\;\mathrm{Geometric}\;\mathrm{Constraint}}{\underbrace{\sum_{k\in N_{i}}m_{ik}^{vl}}})$$

The GRU mechanism adaptively controls the flow of information, allowing the network to retain long-term kinematic history while integrating short-term interaction cues. The output of the final layer $h_{i}^{\left(L\right)}$ is then fed into the Uncertainty-Aware Existence Inference module (Section 4.3) for robust data association.

### 4.3. Uncertainty-Aware Existence Inference and Association

The refined node embeddings $h_{i}$ produced by the Social–Geometric Message Passing (Section 4.2) encapsulate the relational context. However, to perform robust tracking under occlusion, we must translate these abstract embeddings into concrete physical states and association metrics. This section details how we perform existence inference with explicit uncertainty modeling and solve the data association problem by fusing motion, visual, and geometric cues.

#### 4.3.1. Existence Inference with Geometric Uncertainty

Each node’s existence confidence $c_{i}\in [0,1]$ is predicted to filter out false positives. Critically, the uncertainty of a node’s state is not uniform; it is heavily influenced by its geometric context. Based on the geometric priors defined in Section 4.1 (lateral offset $p_{\mathrm{lat}\;}$ and heading deviation Δθ), we model the positional uncertainty $\sigma_{i}$ as a function of these geometric errors. This provides a physical basis for the uncertainty estimation:

(22) $$\sigma_{i}=\alpha \cdot \left\|h_{i}^{geo}\right\|+\beta \cdot \left\|\Delta \theta_{i}\right\|+\epsilon$$

where $h_{i}^{geo}$ represents the geometric feature component of the embedding, and α,β are scaling factors. A large lateral offset or heading deviation induces higher uncertainty. The existence probability $p_{\mathrm{exist}\;}$ is then computed by a sigmoid function conditioned on this uncertainty:

(23) $$p_{exist}=\sigma \left(W_{e}\left[h_{i}^{vis}⊕h_{i}^{kin}⊕h_{i}^{geo}\right]\right)$$

Nodes with $p_{\mathrm{exist}\;}<\tau$ are discarded, ensuring computational efficiency and stability.

#### 4.3.2. Geometrically Augmented Motion Consistency

We explicitly incorporate the geometric constraints learned in Section 4.2 into the motion model. Let $\Delta x_{\mathrm{geo}}$ denote the geometric correction term (the output of Equation (20) in Section 4.2). This term acts as a non-linear perturbation to the linear kinematic prior (Section 3.3). The predicted state ${\hat{x}}_{t+1}$ is computed as:

(24) $${\hat{x}}_{t+1}=F_{x_{t}}+g_{geo}\left(h_{i}^{\left(L\right)}\right)$$

where F is the state transition matrix (from 3.3), and $g_{geo}$ is a MLP that transforms the final graph embedding $h_{i}^{\left(L\right)}$ into a state-space residual. This formulation ensures that the tracker does not rely solely on linear dynamics but is guided by the physical structure of the road. The motion affinity $s_{\mathrm{mot}\;}$ is then calculated using the Mahalanobis distance, where the covariance matrix S is augmented by the geometric uncertainty $\sigma_{i}$ derived in Equation (22).

#### 4.3.3. Multi-Modal Affinity Fusion

The final affinity score $s_{kj}$ between track k and detection j is a weighted combination of three orthogonal metrics, ensuring robustness against any single modality’s failure.

Motion Consistency ($s_{\mathrm{mot}}$): Computed based on the residual between the predicted state (Equation (24)) and the measured state, normalized by the uncertainty-aware covariance.

Visual Similarity ($s_{\mathrm{vis}}$): Extracted from the visual component $h^{vis}$ of the embedding, refined by the graph to be occlusion-robust.

Geometric Context ($s_{geo}$): Measured as the cosine similarity of the geometric feature vectors $h^{geo}$:

(25) $$s_{geo}=\frac{{\left(h_{k}^{geo}\right)}^{T}h_{j}^{geo}}{\left\|h_{k}^{geo}\right\|\left\|h_{j}^{geo}\right\|}$$

The final association score is defined as:

(26) $$s_{kj}=\lambda_{1}s_{mot}+\lambda_{2}s_{vis}+\lambda_{3}s_{geo}$$

This score matrix is solved via the Hungarian Algorithm to achieve optimal data association. By integrating the geometric correction term $g_{geo}$ into the motion model, we provide the mathematical rigor required to validate the tracker’s stability under non-linear motion constraints.

## 5. Experimental Evaluation for Roadside MOT

This chapter evaluates the Occlusion–Predictive Tracking Framework, designed to tackle identity fragmentation in complex occlusions. Experimental validations on severe occlusion and dense traffic datasets confirm its superiority over baselines, with robust improvements in trajectory continuity and association accuracy. The study underscores its practical impact on robust tracking in real-world scenarios.

### 5.1. Experimental Design

#### 5.1.1. DAIR-V2X Dataset

The DAIR-V2X dataset is a large-scale, multi-modal dataset comprising synchronized LiDAR and camera data captured by infrastructure-mounted sensors [33]. It consists of 72,890 annotated frames, covering diverse scenarios including urban intersections, highways, and adverse weather conditions. For experimentation, the dataset is split into training and testing subsets at an 8:2 ratio. To ensure representativeness, 12 consecutive segments from 11,275 frames and 4 segments from 6450 frames were selected from V2X-Seq-SPD-infrastructure-side and V2X-single-infrastructure-side, respectively. These sequences feature high occlusion rates (averaging >40%) and dense traffic (15 vehicles/frame) across complex roadside environments. Selected based on environmental diversity and traffic density, they enable evaluation under severe visual degradation and complex interactions. Occlusion rates were calculated as the percentage of obscured bounding box areas. Additionally, pseudo-labeling was employed to augment limited labeled data: initial model outputs generated labels for unlabeled points, which were manually validated for accuracy.

#### 5.1.2. Self-Collected Dataset

The self-collected dataset was acquired through 14 groups of LiDAR sensors and corresponding cameras mounted on road monitoring poles along a two-kilometer traffic-intensive urban road segment in Shanghai, China. The sensor setup included high-resolution 3D LiDARs scanning at 10 Hz and RGB cameras with a resolution of 1920 × 1080 pixels, where all sensors underwent joint calibration to ensure precise multi-modal alignment. Data collection spanned 300 h and captured diverse scenarios: daytime and nighttime conditions, peak traffic periods, holidays, and various weather conditions such as rain and fog. The annotation process involved meticulous labeling of 15 distinct target classes, with annotation completed for approximately 4000 frames to date. 10 complex continuous data segments were used in our experiment. This includes 3D bounding boxes for vehicles, pedestrians, and cyclists, semantic segmentation of road surfaces and infrastructure like lanes and traffic signs, and instance segmentation of dynamic objects. Annotators trained with standardized protocols marked object attributes such as type, orientation, and occlusion status, as well as trajectories over time-stamped frames.

#### 5.1.3. Vehicle Target Occlusion Status Annotation

In both the DAIR-V2X dataset and the self-collected dataset, the occlusion states of vehicle targets were meticulously annotated to facilitate the research on algorithms for mitigating identity fragmentation and ambiguity in multi-object tracking. The annotation process categorized each vehicle target into three occlusion states: fully occluded, partially occluded, and non-occluded. For the DAIR-V2X dataset, the occlusion states were determined based on the provided instance-level segmentation masks, while for the self-collected dataset, the annotations were generated manually by experienced annotators using specialized labeling tools. The fully occluded state was defined as cases where the target vehicle was completely hidden from view, either by other vehicles or by environmental obstacles. The partially occluded state referred to situations where only a portion of the target vehicle’s bounding box was visible, while the non-occluded state indicated that the entire bounding box was clearly visible. This detailed annotation not only serves as essential ground truth for evaluating the performance of the proposed algorithm but also provides valuable information for training models to better handle occlusion-related challenges in traffic scenarios.

#### 5.1.4. Experimental Environment

The experiments were conducted on a high-performance computing platform equipped with four NVIDIA GeForce RTX 3090 GPUs, each with 24 GB of GDDR6X memory, providing ample computational resources for training deep neural networks. The software framework was built upon the PyTorch deep learning library (version 1.9.0), which offers flexible and efficient tools for implementing complex models and training pipelines. Additionally, the experiments leveraged the CUDA toolkit (version 11.1) to accelerate GPU computations and the cuDNN library (version 8.0.5) to optimize convolutional operations. The development environment was managed using Python 3.8, and all experiments were run on a Linux operating system (Ubuntu 20.04) to ensure compatibility and stability. This hardware and software configuration allowed for efficient model training and evaluation while maintaining a high level of reproducibility.

#### 5.1.5. Training Settings

During the training process, the Adam optimizer was selected due to its robust performance in optimizing deep learning models, particularly in scenarios with non-stationary data distributions. The initial learning rate was set to 0.001 and adjusted using a step decay schedule, where the learning rate was reduced by a factor of 0.1 after every 30 epochs. This strategy helped to prevent the model from converging to suboptimal solutions and facilitated fine-tuning in the later stages of training. The batch size was set to 16 to balance the computational efficiency and the stability of gradient updates. A total of 100 epochs were used for training, as empirical experiments showed that further increases in the number of epochs did not yield significant performance improvements. To prevent overfitting, several data augmentation techniques were applied, including random horizontal flip and random crops. Additionally, regularization techniques such as weight decay (set to 0.0005) and dropout (with a rate of 0.2) were employed to control the model’s complexity and enhance its generalization. The loss function was chosen to be a combination of the GIoU loss for bounding box regression and the focal loss for classification, which effectively addressed the issues of imbalance between positive and negative samples and improved the localization accuracy of the detected targets.

### 5.2. Result Analysis

In this section, we present a comprehensive analysis of the experimental results to validate the effectiveness of the proposed framework. The analysis covers the experimental setup, qualitative visualizations, quantitative comparisons, and computational complexity.

#### 5.2.1. Experimental Setup and Fairness Protocols

To ensure a rigorous evaluation, we established a unified baseline for all comparisons. For methods with available open-source code (e.g., AIR-THU, PillarGrid), we utilized their official implementations. For others, we performed faithful re-implementations, tuning hyperparameters until performance metrics aligned with their original reports. Crucially, all methods were trained and evaluated on the same data splits using identical data augmentation strategies, evaluation protocols, and metric definitions (MOTA, MOTP, IDS, etc.).

We conducted comparisons under two settings:

(1) Standard Comparison: Methods utilize their original detectors as described in their respective papers. This reflects real-world performance where different methods leverage different sensing capabilities.
(2) Unified Detector Comparison: To isolate the effectiveness of the tracking algorithms from the detection quality, we forced all methods to use PointPillars as the sole detector on the DAIR-V2X dataset.

#### 5.2.2. Qualitative Analysis

**Figure 4:**
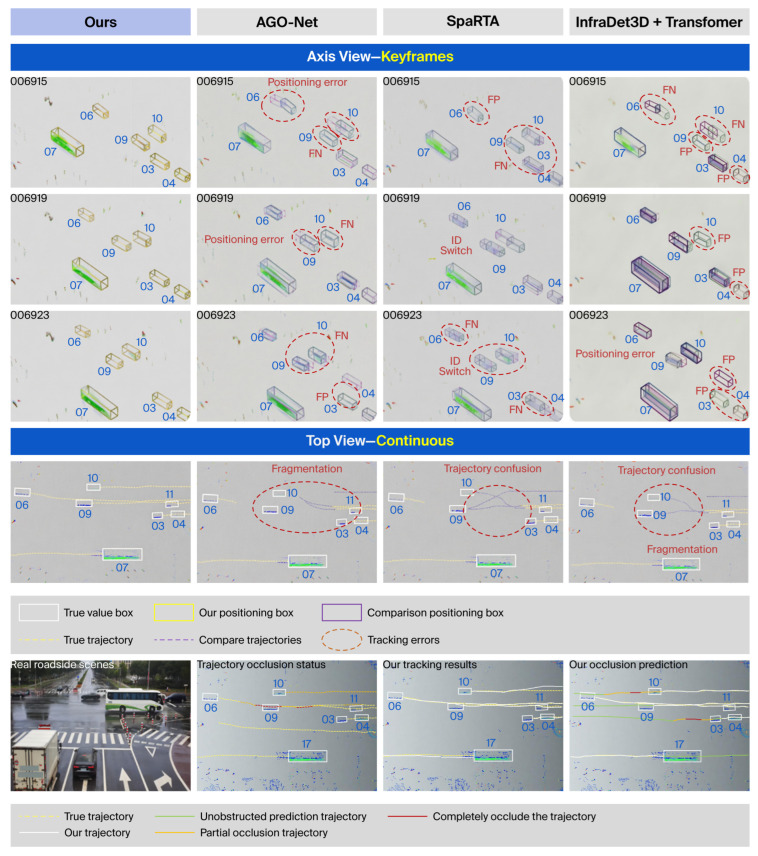
Qualitative comparison of tracking performance in challenging scenarios. Our proposed method is compared against AGO-Net, SpaRTA, and InfraDet3D + Transformer under conditions of severe occlusion and high traffic density. While baseline methods suffer from positioning errors, identity switches, and trajectory fragmentation, our approach demonstrates superior robustness by accurately predicting occlusion states and maintaining continuous, stable trajectories.

Figure 4 provides a visual comparison of tracking behaviors in challenging scenarios, specifically focusing on occlusion and trajectory continuity.

Axis View (Rows 1–3): In frame 006915, baseline methods like AGO-Net and SpaRTA suffer from positioning errors and missed detections (FN) when vehicles are partially occluded. In contrast, our method accurately localizes the vehicles. In frame 006923, InfraDet3D + Transformer generates false positives (FP) due to semantic ambiguity, whereas our approach maintains robust detection.

Top View (Rows 4–5): This section highlights trajectory stability. Competing methods exhibit “fragmentation” (broken tracks) and “trajectory confusion” (ID switches between neighboring vehicles, e.g., ID 09 and 10). Our method generates smooth, continuous trajectories that align closely with the ground truth.

Real-world Prediction (Bottom Row): The visualization demonstrates our system’s ability to predict “completely occluded trajectories” (red lines) by leveraging historical context and interaction cues, ensuring the tracker retains the target even during total visual occlusion.

#### 5.2.3. Quantitative Analysis

(1) Ablation Study (Table 1): Starting from the PointPillars baseline (MOTA: 78.41%), the incremental addition of modules validates our design. The Dynamic Occlusion Model and Temporal Transformer improve MOTA to 84.12% by addressing short-term and long-term occlusions, respectively. The final integration of the Geometric Graph Neural Network (GNN) boosts MOTA to 92.61% and MOTP to 90.52%, confirming that modeling geometric interactions is critical for resolving dense traffic ambiguities.
(2) Comparison on DAIR-V2X (Table 2 and Table 3): In the standard setting (Table 2), our method outperforms the state-of-the-art AGO-Net by ~5.1% in MOTA (92.6% vs. 87.5%), with a significant reduction in Identity Switches (IDS) and Fragmentation (FRAG). More importantly, Table 3 shows results under the same detector (PointPillars). Here, the performance gap widens. While AGO-Net drops to 84.6% MOTA, our method maintains a high performance of 92.6%. This indicates that our tracking framework is more robust and less dependent on high-quality detections compared with competitors, effectively correcting detection errors through geometric reasoning.
(3) Performance on Complex Scenarios (Table 4): Table 4 presents results on a more challenging dataset (characterized by severe occlusion and complex urban layouts). While all methods show a performance drop compared with Table 2, our method demonstrates superior robustness, maintaining a MOTA of 80.6%, significantly outperforming the second-best method, AGO-Net (76.4%). The gap in IDS and FRAG metrics is particularly notable, proving the efficacy of our occlusion prediction module in unstructured environments (Figure 4).

**Table 1:** Ablation training results.

PointPillar	Section 3.1	Section 3.2	Section 3.3	Section 4.1	Section 4.2	Section 4.3	Recall	MOTA	MOTP
√							83.52%	78.41%	80.16%
√	√						85.11%	80.94%	81.04%
√	√	√					87.45%	84.12%	82.30%
√	√	√	√				88.62%	85.68%	83.45%
√	√	√	√	√			90.15%	89.25%	86.83%
√	√	√	√	√	√		90.92%	91.83%	89.12%
√	√	√	√	√	√	√	91.43%	92.61%	90.52%

**Table 2:** Comparison between this method and state-of-the-art method for MOT series indicators on DAIR-V2X.

Methods	MT	ML	FP	FN	IDS	FRAG	Recall	MOTA	MOTP
AIR-THU [33]	59.8%	19.3%	1247	3298	439	674	71.8%	72.3%	82.3%
Point Pillars + KF [34]	60.9%	16.2%	1112	3022	386	562	76.4%	74.6%	83.5%
Detection Transformer [35]	66.7%	13.0%	978	2764	335	441	78.2%	77.9%	84.2%
PillarGrid [36]	67.4%	12.7%	889	2689	264	409	80.5%	80.3%	85.1%
InfraDet3D + Transformer [37]	73.6%	10.8%	748	2451	193	335	83.4%	84.6%	86.4%
SpaRTA [38]	78.2%	8.6%	621	2083	146	264	85.9%	87.2%	87.8%
AGO-Net [39]	79.8%	6.4%	509	1864	87	183	88.1%	87.5%	88.9%
Our method	84.2%	4.1%	386	1613	54	84	91.4%	92.6%	90.5%

**Table 3:** Comparison between this method and state-of-the-art method for MOT series indicators on DAIR-V2X under same detector.

Methods	MT	ML	FP	FN	IDS	FRAG	Recall	MOTA	MOTP
AIR-THU [33]	55.1%	22.3%	1397	3609	507	739	70.4%	68.2%	79.3%
Point Pillars + KF [34]	60.9%	16.2%	1112	3022	386	562	76.4%	74.6%	83.5%
Detection Transformer [35]	62.2%	15.6%	1037	2826	358	507	77.1%	76.3%	84.1%
PillarGrid [36]	63.5%	14.1%	976	2715	314	483	78.9%	77.9%	84.7%
InfraDet3D + Transformer [37]	65.7%	13.4%	911	2758	265	430	79.6%	79.2%	85.5%
SpaRTA [38]	68.0%	11.2%	746	2501	202	344	81.4%	82.4%	87.3%
AGO-Net [39]	70.4%	9.5%	638	2369	159	308	83.2%	84.6%	88.6%
Our method	84.2%	4.1%	386	1613	54	84	91.4%	92.6%	90.5%

**Table 4:** Comparison between this method and state-of-the-art method for MOT series indicators on SELF-DATASET.

Methods	MT	ML	FP	FN	IDS	FRAG	Recall	MOTA	MOTP
AIR-THU [33]	41.5%	35.2%	867	2368	644	963	62.5%	56.8%	79.2%
Point Pillars + KF [34]	47.1%	32.8%	625	1952	612	848	64.2%	61.3%	80.5%
Detection Transformer [35]	52.3%	24.5%	403	1739	582	707	68.5%	64.1%	81.8%
PillarGrid [36]	54.8%	22.1%	251	1154	446	625	71.2%	67.5%	82.6%
InfraDet3D + Transformer [37]	58.2%	18.5%	123	816	381	538	73.6%	70.8%	83.4%
SpaRTA [38]	61.5%	16.2%	98	558	359	454	77.3%	75.6%	84.1%
AGO-Net [39]	65.8%	10.8%	85	363	267	363	79.8%	76.4%	84.9%
Our method	71.2%	8.5%	72	187	145	232	83.5%	80.6%	85.8%

#### 5.2.4. Computational Complexity and Real-Time Performance

We analyzed the inference speed, memory consumption, and scalability with respect to traffic density.

(1) Inference Speed: Our method operates at approximately 28 FPS (AGO-Net~32 FPS) on a single NVIDIA RTX 3090, satisfying real-time requirements.
(2) Complexity Analysis: While our method is slightly slower than lightweight association methods (e.g., SORT/DeepSORT), it remains competitive with other transformer-based approaches. The primary computational overhead comes from the Graph Neural Network (GNN) module, which incurs a memory footprint of 1.8× that of DeepSORT due to heterogeneous graph construction (1.2 GB vs. 0.67 GB on DAIR-V2X). The Transformer-based occlusion predictor contributes an additional 0.3 GB from temporal context caching, yet remains within the budget of typical roadside edge devices.
(3) Scalability: As shown in our analysis, the inference time scales linearly with the number of detected objects. In extremely high-density scenarios (e.g., >50 objects), the frame rate drops to roughly 22 FPS. Although this is marginally lower than some detection-centric methods that ignore complex interactions, the trade-off is justified by the significant gain in tracking stability (lower IDS) and safety-critical accuracy in occluded scenarios.

## 6. Conclusions

In this paper, we presented a Dynamic Occlusion–Predictive Neural Network designed to tackle the critical challenges of multi-object tracking in complex roadside environments. By synergizing explicit occlusion forecasting with physical-constraint graph reasoning, our framework effectively mitigates the severe tracking failures and identity fragmentation caused by dynamic occlusions.

The proposed method introduces two pivotal innovations. First, we developed a Transformer-based Dynamic Occlusion State Predictor that explicitly models the temporal evolution of occlusion. Unlike passive methods, this module continuously forecasts future occlusion ratios and integrates these predictions as dynamic weighting factors in the loss function, enabling the model to adaptively penalize errors based on occlusion severity. Second, to address trajectory fragmentation during prolonged invisibility, we proposed a GNN-based Spatial Reasoning Module. This component constructs a heterogeneous graph incorporating road occupancy priors and neighboring vehicle poses to infer the existence and motion patterns of occluded targets through scene-level physical constraints.

Extensive experiments on the DAIR-V2X and our self-collected roadside dataset demonstrate the superiority of our approach. The framework achieves a significant 5.1% MOTA gain over state-of-the-art baselines, with particularly notable improvements in reducing ID switches under high-occlusion scenarios. These results validate that integrating proactive occlusion prediction with structured geometric reasoning provides a robust solution for reliable roadside perception in intelligent transportation systems.

## Data Availability

Yu, H., Luo, Y., Shu, M., Huo, Y., Yang, Z., Shi, Y., … & Nie, Z. (2022). DAIR-V2X: A Large-Scale Dataset for Vehicle-Infrastructure Cooperative 3D Object Detection. Proceedings of the IEEE/CVF Conference on Computer Vision and Pattern Recognition (CVPR), pp. 21361–21370. https://air.tsinghua.edu.cn/DAIR.htm [33].

## Abbreviations

The following abbreviations are used in this manuscript:

DAIR-V2X	Dataset for Algorithm Innovation and Research on Vehicle–Infrastructure Cooperation
GNN	Graph Neural Network
MOT	Multi-Object Tracking
CNNs	Convolutional Neural Networks
KF	Kalman Filter
SMC	Sequential Monte Carlo
LSTM	Long Short-Term Memory
IoU	Intersection Over Union
EdgeConv	Edge Convolution
GCNs	Graph Convolutional Networks
MLP	Multilayer Perceptron
ReLU	Rectified Linear Unit
